# Guyanese Women's Experiences of Invisibility in Health Care in England

**DOI:** 10.3389/fsoc.2020.00023

**Published:** 2020-04-09

**Authors:** Helena Ann Mitchell

**Affiliations:** The Open University, Milton Keynes, United Kingdom

**Keywords:** participatory, action, intersectionality framework, diabetes, community

## Abstract

This article explores the opportunities for strengthening participatory action research (PAR) through an intersectionality framework. In 2015, I completed a Ph.D. study into the lived experiences of migrant Guyanese women, living in England, when seeking diagnoses and treatment for Type 2 diabetes. Group storytelling acted as a lens for the women to talk about how they tried to obtain a diagnosis, in addition to their migratory experiences. Both PAR and intersectionality encourage participant collaboration and community engagement of oppressed groups. The article concludes that the PAR study would have been enhanced by overlaying it with an intersectionality framework. The argument is presented that by doing so the women's accounts in the research study would have been privileged more and activism encouraged in bringing about change to current practices and avoiding perpetuating existing oppressions. The Ph.D. study methodology was based on Koch's interpretation of PAR. In this, PAR is used where the focus is on participation of all stakeholders toward reform and change. It is seen as a social, practical and collaborative process where building relationships with participants is crucial. Intersectionality acknowledges the potential for “black” and other women of color to not remain on the margins but to challenge the traditional biomedical model of health care delivery. Implementing an intersectional approach to the data generation and analysis would have acknowledged power dynamics (i.e., privilege and oppression) and help to identify potential gaps in diabetic provision which are currently invisible or inequitable due to interventions designed to meet the needs of a homogeneous White middle class society.

## Background

This article explores the opportunities for strengthening participatory action research (PAR) by overlaying that methodology with an intersectionality framework. The basis for this proposition arises from consideration of the effect of overlaying such a framework on a Ph.D. thesis published by me in 2015. This original study explored the lived experiences of migrant older “Black” Guyanese women trying to obtain a diagnosis for Type 2 diabetes through the UK health care system. The PAR methodology was chosen as the most appropriate approach for giving voice to those whose voices were largely unheard in a society where they faced racist issues and oppressive practices as they tried to access health care for a long term condition. Having learnt about intersectionality some years later I reflected on the potential for an intersectionality framework to offer greater insights into the research study. I now feel that overlaying an intersectionality framework could have enhanced and enriched the study especially the data analysis element. This article proposes that an intersectionality framework would provide researchers with an additional tool set when using PAR methodology. The question to be explored in this paper is how could an intersectionality framework have added to those insights?

## Understanding the Purpose of Intersectionality

Many terms are used to define intersectionality. Crenshaw (1989), a key exponent of intersectionality, sees it as being structural and political. Structural intersectionality defines how ideology, class, gender, and racial positioning influence people's interaction with mainstream services. Political intersectionality explores the conflicting agendas of antiracism and feminism (Crenshaw, 1989). Collins (1991) also published extensively on the topic. More recent authors like Bowleg (2012) consider intersectionality to not be a theory or empirically tested. Instead she frames the term within public health and describes it as an “analytical framework that departs from a traditional biomedical model, bio behavioral and psychosocial paradigms that have shaped medicine, public health and other social sciences” (Bowleg, 2012, p. 1270). Lewis (2013, p. 873) considers how intersectionality was “greeted with hope and applause because of both its theoretical scope and its empirical inclusivity. It welcomed the margins to the table of theory making by reconciling the split between theory and experience—or, more precisely, by suggesting that experience could be the ground of theory making.” It seems intersectionality as a framework has the potential to disentangle the many factors that influence minority people's lives leading to disadvantage, varying levels of oppression, inequalities and inequities they face.

It is important to admit that implementing an intersectional approach is still relatively new in the UK and seldom seen in health care research. Criticism has been levelled at it for being poorly defined and inadequately operationalised (Mens-verhulst and Radtke, 1991), and there has been a debate among scholars regarding the implementation of this approach. Some have suggested using it for complex research designs while others focus more toward narrative work (Mens-verhulst and Radtke, 1991). Furthermore, it has also been suggested that language itself is limited in the explanation of the concept of intersectionality (Gunderson and Cochrane, 2012).

It is noted that intersectionality has many advantages as postulated by Mens-verhulst and Radtke (1991) and Gunderson and Cochrane (2012) as it considers the power dynamics of privilege and oppression and demonstrates how structural level services maintain inequality in ways other theoretical models have failed to Bowleg (2012). I therefore believe that if intersectionality had been used as a framework in the PAR study (Mitchell, 2004) the focus would have shifted more onto the health and social inequalities and the oppressive practices that the women faced within society. When the women's experiences are dissected, as evidenced in the PAR data, it is astounding to see the complexity of the intersecting variables in terms of race, class and identity as they tried to cope with a debilitating chronic illness (Hankivsky et al., 2010). These multiple disadvantages were perceived as preventing them from accessing the relevant services as highlighted by Kapilashrami and Hankivsky (2018) who argue that systemic racial issues such as equal accessibility and distribution of resources can affect certain groups like African-Caribbean immigrant women.

The next section in this paper gives insight into the study including an overview that details the sample group, methodology, analysis, and findings. The intersectionality framework will then be applied to these various components illustrating how the study and its findings could have been enhanced. To clarify the two positions in this paper, intersectionality is rooted in Black feminism and critical race theory whereas PAR emerged as a liberationist practice aiming to redress imbalances of power. There is a distinct difference in the two approaches which will be further explored.

## The Par Study

In setting the context for this paper it is important to briefly describe the original PAR study (2010–2015) and provide background information about the women and why they were chosen. The rationale for choosing the PAR methodology is included, along with the insights gained into the women's lived experiences as they grappled with accessing appropriate services for their long term condition. PAR as a methodology within this context, is primarily used as a qualitative process to provide insight into the lived experience.

## Ethical Approval

Ethical approval for the study was provided by the University of Surrey Ethics committee and conducted within their ethical framework (EC/2010/08/FHMS). The study did not involve life threatening situations, emotional discomfort or distress. Participants were informed of their right to withdraw at any point or refuse to answer any of the questions. No identifying details of them were recorded and pseudonyms were used for transcribed interviews and reporting findings. Participants provided written and informed consent prior to commencement of the interview process. Datasets were generated and analyzed in this study.

The chosen sample group were Guyanese women, born and raised in Guyana whom had lived for an average of 40 years in England. They came from middle class backgrounds in Guyana, previously known as British Guiana, which gained independence from the United Kingdom in May 1966. The country is situated on the mainland of South America however it is normally associated with the Caribbean due to its British colonial past. Politically, Guyana became a founding member of CARICOM (the Caribbean community) and an important part of the single market and single economy of the other Caribbean member states. Guyana was an important contributor to the influx of “West Indian” immigration to the UK commonly referred to as the “Windrush Generation.”

The women in the study were totally unaware that they had Type 2 Diabetes even though they had accessed health care services on several occasions. As a consequence this lack of recognition of the signs and symptoms associated with this long term condition resulted in the women acquiring many of the complications of diabetes. In applying the intersectionality framework it would have provided the ability to recognize that the women's experiences in not accessing relevant services may have been influenced by a number of other factors such as ethnicity, discrimination and marginalization. Consequently, Black women experience disempowerment on several interacting levels such racism, oppression, power imbalance, and discrimination when accessing health care (Crenshaw, 1989).

Whilst PAR as a methodological approach (Dick, 2004; Baum et al., 2006; Bradbury, 2015) gave voice to the women, there being limited literature relevant to this migrant group, it was distinctive as a research methodology due to its focus on collaboration, political engagement, explicit commitment to social justice, and emancipatory action (Brydon-Miller et al., 2015). Distinguishing features tended to relate to it being community based and driven, its sole purpose being to generate knowledge or understanding that leads to reform and brings about change (Baum et al., 2006; Reason and Bradbury, 2006; De Chesnay, 2014). Relationship building is central to the process and participants set the agenda and take action to bring about personal change in their lives.

The philosophical underpinning in this study was based on the work by Habermas (1972, 1984) that influenced knowledge creation. His theoretical thinking was focused on emancipation and, like Friere (1970), Habermas's social theory advanced the goals of human participation, communicative action, and entailed a theory about dialogue as individuals meet in conversation. Habermas (1984) argued for the democratization of research and encouraged those excluded from the process to have a voice. Consistent with Habermas's worldview is that PAR is motivated by a desire to secure authentic information (new knowledge) about people and situations that embraces experience as a source of legitimate knowledge.

The participatory action research methods of data generation used in this study consisted of storytelling interviews, focus groups and a research journal. The PAR approach guiding this work follows the processes of “looking, thinking, and acting” as a systematic cyclical action (Koch and Kralik, 2006; Koch, 2015) which has been used with many groups living with a chronic illness. The PAR approach endorses the centrality of storytelling in health care to transform peoples' lives as participants bring their own knowledge to the research and constant validation of this knowledge is the cornerstone of the PAR process. On a one to one basis, participants were able to tell their own story in whichever way they wished in interviews and were asked: tell us what it is like living with diabetes? Interviews took place in their own homes. In the focus groups, the participants volunteered, set their own agenda based on their needs, share experiences, learn from each other and decide on the actions so that voices come from the grass roots. For the researcher keeping a journal was important because it entailed reflexivity, aided reflection and encouraged the researcher to think about her reactions to the participants and record feelings (Waterman, 2013). Equally the women reflected during story telling interviews and within group sessions and shared their reflections with each other.

The analysis of the data consisted of the “*looking,” “thinking,” and “acting”* process as information gathering (Koch and Kralik, 2006). The looking phase occurs as the participants shared the resources and information about diabetes during this process. *Thinking* gives the individual/group time to reflect, to make sense of what is happening and to engage in dialogue with others in the group if they so desire. When “a*cting,”* individuals consider options or choices available to them toward reform. The transcribed interviews were analyzed with the participants input and a storyline developed. The subsequent storyline was analyzed concurrently by the researcher and the participants making their stories accessible for reflection, discussion and the actions the women wanted to take as a result of reflections. Individual actions initiated by the women usually referred to a lifestyle change such as doing more exercise or the selection of food alternatives that comprise healthy eating. These processes are interactive and cyclical as mentioned earlier. The participants drive *action* when they are ready. The journal data constituted what was going on whilst researching as self-awareness developed. Reflection in PAR is crucial for both the researcher and the participants as they rethink their position, discover new ways of being, acting and doing, and deal with the issues that they face during the research process. (Schon, 1983) model of reflection guided the reflection.

In summary PAR is less committed to racial dominance but favors marginalized groups. In contrast intersectionality can create a connection around shared experiences of discrimination and marginalization (Carbado et al., 2013). It places marginalized groups at the center of research, privileges the perspectives of groups who have been dominated by white people and have been subjected to practices that perpetuate racial domination and reproduce social inequality (Douglas, 2017).

## Linking Intersectionality to Par

Intersectionality would have privileged the accounts from the Guyanese women in more detail and encouraged them in activism to bring about change in current practices. Implementing an intersectional approach to the data generation and analysis would have acknowledged power dynamics such as privilege and oppression. This would help identify potential gaps in diabetic provision currently invisible or inequitable resulting from interventions designed to meet the needs of a homogeneous White middle class society. Changes in legislation and policy expected health services to address inequalities (Department of Health Social Care, 2012; Marmot, 2013; Bircher and Kuruvilla, 2014) but did not do so for marginalized groups. Recognizing previously excluded populations and identifying structural factors such as health/social care policies and health care practices that maintain inequality needs to be addressed. An intersectional approach is ideally suited to exploring health inequalities as the approach challenges the existing power dynamics within society.

When analyzing the findings it emerged that the women had developed a more nuanced understanding of the effects of migration on their experiences of being Guyanese and having diabetes. They realized the food they ate was not appropriate for an individual who had diabetes. Access to services for diagnosis and treatment of their long term condition was another important finding that impacted on their lives. However, using the intersectionality framework would have represented a different approach in understanding the complexities of health inequities. The framework would have provided awareness of how specific categories such as socioeconomic class, race, ethnicity, or a migration background should not be analyzed separately but as interacting factors (Kapilashrami and Hankivsky, 2018).

The focus groups gave the women the women the confidence to share their thoughts and feelings with each other and reported an increase in managing their long-term condition. They had lived with diabetes for several years prior to the focus groups and reported that the exchange of information was useful and thought provoking. The women's storytelling interviews and data gathered in the focus groups suggested that the “home” or traditional food they ate after migrating helped retain their cultural identity. When they developed Type 2 diabetes such food was considered unhealthy but in the groups, the women reconsidered lifestyle changes such as exercise and healthy eating more important. The access to the general practitioner's (GP) service was considered an issue during the interviews and group sessions and one indicated how it felt, “I made every effort to access the GP by contacting him regularly yet it took me a long time before I was diagnosed with the condition even with a family history.” They wondered whether the delay in being diagnosed with diabetes was due to discrimination in health care delivery. Vera told the group she was still enraged by her treatment where she was passed from one specialist to another. Eventually Vera said “she was seen by a dermatologist complaining about hives because the hives irritated her and caused her to scratch instantly.” She reported that the doctor looked at her “in almost disgust” and said “look you may have a few health problems but by and large you are healthy.” Vera said “I just wanted to slap him.” On reflection the quality of the women's lives was being undermined by the health care system but this factor was not fully addressed when writing up the study. However, the potential of intersectionality has now heightened my awareness of how the institutional systems of health care use its structures to advance health inequity in marginalized groups. The women did not experience the same amount of health benefits as white women. Racism and sexism could not be easily disentangled as they grappled with both at the same time.

Surprisingly the PAR study enabled the women to adjust to more modified diets, take more regular exercise and regularly check their glucose levels. They were surprised at the portion sizes of food they should be eating: it took them time to recognize that eating large quantities of food high in fats and carbohydrates needed to be avoided. Bea stated “when you eat little portions you can control the sugar level and I thought I must do that.” Sharing information led to an increase in knowledge about the condition to consider what was the appropriate portion size however as they attempted to modify their diet, they faced dilemmas about their favorite foods. Pam said “Guyanese ate too much home food.” The yearning and craving seemed to deepen when they could not have them. The women concluded that they ate big portions of foods in a diet that is not good for people with diabetes. The women developed knowledge and understanding that their preferred diet may not be appropriate to their needs. They were ready to change their culturally influenced diet to a more varied one resulting in a behavioral change. This occurred as a result of the collaborative interactions and support offered to each other in the PAR groups.

## Discussion

Type 2 Diabetes is on the increase (Diabetes UK, 2015) but when compared with women in the general population, diabetes is five times more likely in Pakistani women, at least three times more likely in Bangladeshi and Black Caribbean women (Diabetes UK, 2012; Tillin et al., 2012). Despite attempts to seek a diagnosis for their condition the women in this study felt marginalized and powerless. The delay in being diagnosed left the women feeling confused, lonely, and ignored. Most wanted a confirmed diagnosis before they felt confident in making lifestyle changes (Odette et al., 2004).

The women wondered whether the delay in being diagnosed with diabetes was a consequence of them being “Black.” Did they imagine it or did they experience discrimination in health care delivery? In the group sessions they talked about racism having the power to disrupt a person's life. Back (2005) postulates that racism is typically defined as a form of spatial and territorial form of power that aims to secure a particular territory which the other claim as their own. Being “Black” in a white society can be perceived as only “whiteness” having value according to Alleyne (2002) and White (2006) as Black people aim to seek recognition of their own value and dignity and oppose racial stereotyping.

Szczepura (2005) reviewed evidence on access to health care by ethnic minority populations and highlighted that it has not been possible to develop a UK overview of disparities in access to the service or to monitor these nationally because the data does not provide sufficiently detailed information. She believed that “simply providing an equal service cannot ensure access to care for all people regardless of their religion, culture or ethnic background. Therefore, having access to that you will be treated with respect” (Szczepura, 2005, p. 142) and having the confidence the services should be relevant, timely and sensitive to the person's needs.

The women had identified in the study the kind of diabetic service that was preferable, one that was consultative with a holistic approach to the care and treatment. They believed the “one size fits all model” currently provided for all individuals with Type 2 diabetes was not culturally appropriate for those with specific beliefs and needs because it did not address cultural variations, underlying motivations, preferences and behaviors. If intersectionality had been integrated in the study the women's suggestion would have been shared more widely with the community to activate change in local diabetic services for minority groups.

The women realized their identity was strong as they shared experiences and spoke with pride about their home country and what it was like to be Guyanese. They had left Guyana many years ago, retained their identity as they grappled with oppression and marginalization but the experiences of discrimination and race had re-emerged as they tried to obtain a diagnosis for diabetes. I felt this issue was not fully addressed in the PAR study and possibly glossed over. Crenshaw (1989) explains that by considering social identities (e.g., race, gender, social class, and religion) which converge and interact, a different socially constructed view is forwarded that accounts for oppression, power imbalance, and discrimination.

On reflection, an intersectionality framework would have enhanced the body of the PAR study. Integrating intersectionality with PAR in a disadvantaged community would have enabled a fuller understanding of health disparities (Kelly, 2009). I am fully conversant with the strengths of using PAR to restore to ordinary people the capacities of self-reliance and the ability to manage their own lives—to “sharpen their minds.' It aims to solve real problems within marginalized communities (Reason and Bradbury, 2006). The primary issues are about creating change, encouraging reflexivity, and influencing policy decisions.

Whilst there are obvious strengths in applying the intersectionality framework, PAR has its strengths too as much of its work has been based on Paulo (Friere, 1970) consciousness raising and critical awareness through practice and participation leading to more community-based interventions. (Friere, 1970) was a leading and inspirational educationalist in South America in the 1950s who researched disenfranchised students and participants to place life-transforming capabilities in their hands. His research falls into the category of critical emancipation that provides evidence from a social perspective that working collaboratively alongside people enables them to develop a new awareness of self which can then respond to change (Friere, 1970). Within this context of Friere's work, the women in the PAR study had responded to change favorably leading to significant changes in their lifestyle as they became more assertive with the health care team in relation to their care and treatment of their diabetic condition.

Intersectionality may have some similar features to PAR as highlighted by Hankivsky et al. (2010) who also claim that intersectionality focuses on partnership, participation, and reflexivity. This view held by Hankivsky is not too dissimilar to those emphasized by proponents of action research such as Dick (2004), Kemmis and McTaggart (2005), Kidd and Kral (2005), Reason and Bradbury (2006), Bradbury (2015), who describe the components of PAR as participation, collaboration between the researcher and participants and reflexivity. The PAR study privileged the voices of one particular racial group about a particular health condition but less on a health disparity among “black” women *per se*. PAR in this context captured the discriminated community's reality at a micro level as the women shared their discriminatory practices and sought help for type 2 diabetes.

As the women engaged in the PAR process, they felt listened to during the story telling interviews as they reflected on their lives. They increased their knowledge and understanding about their diabetes and tried to take control of it by acquiring information although that was rather limited and fragmented. There was an impetus to change their lifestyles, recognize the physical complications of diabetes and become more assertive when engaging with health care practitioners. A core epistemological assumption of intersectionality is that knowledge development is from the perspective of the oppressed (McCall, 2009; Sallah, 2014) and it is a powerful concept for explaining inequality, social determinants of health and power structure.

The women reflected on how connecting with the diaspora gave them the strength and impetus to change their lifestyle within the group sessions in the study. They became more involved in their local communities and discussed how local healthcare practice could be improved regarding diagnosis and treatment of diabetes. This could be interpreted as activism on a micro scale showing early indications of intersectionality as they strived to bring about change with others having similar long-term conditions.

## Conclusions

Intersectionality has been successful because it acknowledges the potential for “black” and other women of color not to remain on the margins by challenging the micro and macro systems in society. I recognize from writing this paper how my own understanding of intersectionality as a research framework for a study of disadvantaged groups has been enriched. It acts as reminder that racism, sexism, social and health inequalities exist in the UK for Black and minority ethnic (BME) communities. The women in this study have provided some evidence for this. I have argued that intersectionality as a framework could have further enhanced the original PAR study as it sees change more in terms of activism by reaching out to disadvantaged communities and helping to avoid perpetuating existing oppressions. The Ph.D. research project using PAR methodology enabled participants to give voice to their concerns in a way that exceeded the expectations of the participants and the researcher. I would conclude that overlaying an intersectionality framework to the original PAR study would have given the work an improved understanding of health disparities. As a result I have started to incorporate it into current research projects as a means of continuing to improve the impact the PAR methodology can have.

## Data Availability Statement

The raw data supporting the conclusions of this article will be made available by the authors, without undue reservation, to any qualified researcher.

## Ethics Statement

The study received approval from the University of Surrey EC/2010/08/FHMS.

## Author Contributions

The author confirms being the sole contributor of this work and has approved it for publication.

### Conflict of Interest

The author declares that the research was conducted in the absence of any commercial or financial relationships that could be construed as a potential conflict of interest.
